# Enhanced sensitivity to punctate painful stimuli in female patients with chronic low back pain

**DOI:** 10.1186/1471-2377-12-98

**Published:** 2012-09-21

**Authors:** Christian Puta, Birgit Schulz, Saskia Schoeler, Walter Magerl, Brunhild Gabriel, Holger H W Gabriel, Wolfgang H R Miltner, Thomas Weiss

**Affiliations:** 1 Department of Sports Medicine and Health Promotion, Friedrich Schiller University, Wöllnitzer Strasse 42, Jena, D-07749, Germany; 2 Center for Interdisciplinary Prevention of Diseases related to Professional Activities, Friedrich Schiller University, Jena, D-07743, Germany; 3 Department of Biological and Clinical Psychology, Friedrich Schiller University, Jena, D-07743, Germany; 4 Department of Neurophysiology, Center for Biomedicine and Medical Technology Mannheim (CBTM), Ruprecht Karls University Heidelberg, Mannheim, 68167, Germany

**Keywords:** Chronic Low Back Pain (CLBP), Mechanical pain thresholds, Pinprick hyperalgesia, Allodynia

## Abstract

**Background:**

Chronic low back pain (CLBP) has been shown to be associated with various pathophysiological changes at several level of the sensorimotor system, pointing to a general hypersensitivity in CLBP patients. The aim of the present study was to investigate signs of generalized mechanical pain hypersensitivity in CLBP patients on the hand and on the painful site of the back.

**Methods:**

Pinprick stimulation according to a validated standardized quantitative sensory testing protocol was used in 14 female CLBP patients and 14 healthy controls (HC) matched for sex and age. Stimulus response functions to pinprick stimulation on the skin were examined at the affected back and reference sites (hand palmar and hand dorsum). Data from CLBP patients were compared with HC and with reference data from the German Research Network on Neuropathic Pain.

**Results:**

We found significant differences in the stimulus response functions between CLBP patients and HC. Pain ratings to the pinpricks were increased for low and moderate pinprick stimuli in CLBP patients. Importantly, this kind of specific pinprick hyperalgesia was found not only for the affected body site (back), but also for the remote reference sites (hand dorsum and hand palmar).

**Conclusions:**

We interpret our results as pointing to changes in the nociceptive processing in CLBP at higher levels of the neuraxis, possibly thalamus and/or attentional control, rather than changes of spinal processing. Alternatively, there might be a higher vulnerability to noxious stimulation in CLBP patients.

## Background

Chronic low back pain (CLBP) is one of the major health problems in industrialized countries with costs of US$ 100–200 billions a year [1]. Beyond this economic burden, CLBP patients suffer from reduced quality of life, and loss or limitations of employment. Many also complain of psychopathologies such as depression, anxiety, loss of social activities etc. [2,3]. However, pathophysiological changes in CLBP are still enigmatic and call for additional efforts to demystify the underlying pathophysiology, potentially allowing progress to be made towards successful mechanism-based treatment strategies.

CLBP is associated with several pathophysiological changes at various level of the sensorimotor system. At the cortical level, CLBP is associated with functional reorganization in somatosensory and motor regions [4-9]. Changes have also been observed on a behavioural level, namely in attentional control [10,11], during stimulus anticipation [12,13], and in motor behaviour [14-16]. Furthermore, mechanoreceptive [17] and proprioceptive perception is reduced in CLBP [18-20]. However, there are only a few studies using quantitative sensory testing (QST) in CLBP patients. Blumenstiel et al. [21] compared QST profiles of fibromyalgia patients with those of CLBP patients with a focus on fibromyalgia. Investigating QST of the hand and the back, they found significant changes on the backs of CLBP patients with an increased threshold for vibration and a reduced threshold, i.e. hypersensitivity for pressure pain. Their data demonstrate changes to pain thresholds in CLBP patients that might be interpreted as generalized pain hypersensitivity. These authors did not specifically investigate thresholds at the painful site of CLBP patients but used sites similar to the examination in patients with fibromyalgia. Therefore, they are unable to assess the amount of hypersensitivity with respect to their primary syndrome. In line with the above-mentioned generalized pain hypersensitivity, CLBP patients exhibit lower perception thresholds, lower pain thresholds, lower pain tolerance values, and reduced habituation compared to healthy controls [22-24]. Furthermore, the results of an EEG mapping study indicate enhanced perceptual sensitization and enhanced processing of the sensory discriminative aspect of pain in CLBP patients [25].

The aim of the present study was to investigate signs of generalized mechanical pain hypersensitivity in CLBP patients on the hand and on the painful site at the back. We hypothesized that CLBP patients would exhibit increased sensitivity to noxious punctate mechanical stimuli on both the paraspinal lumbar area and the dorsal and palmar aspects of the hand compared to matched healthy controls.

## Methods

### Participants

Fourteen female chronic low back pain (CLBP) patients and fourteen pain-free healthy controls (HC) participated in this study. CLBP patients (for detailed characteristics - see Table 1) met the following criteria: 1. A minimum six month history of low back pain; 2. pain had been classified as ‘non-specific low back pain’ (no indicators of nerve root problems, e.g. unilateral leg pain, radiating to foot or toes, numbness and/or paraesthesia; straight leg raising test induces leg pain); 3. magnetic resonance imaging (MRI) of the spine showed only age-related changes, but no spinal disorders or disc pathology; 4. no psychiatric disorders and no disease associated with small fibre pathology (e.g.; diabetes mellitus) according to clinical anamnesis. All participants were screened for eligibility by a female clinician (B.S.). B.S. instructed the subjects a week before investigation not to take any analgesic medication for at least 48 hours before the examination. Before examination, subjects confirmed they had not taken any medication during the last 48 hours. All participants were right handed and all were employed. The local ethics committee of the University of Jena approved the whole procedure. Participants gave written informed consent, in compliance with the Declaration of Helsinki.

**Table 1:** Characteristics of the female chronic low back pain (CLBP) patients and female healthy controls (HC)

**No.**	**Age (years)**	**Duration of pain (month)**	**VAS**^**QST**^	**NRS**^**4average**^	**NRS**^**4max**^	**BDI**	**RDQ**	**Reported medications (as needed)**
1	52	360	5.8	4	8	11	15	No medication at all
2	54	168	5.1	5	7	12	6	Diclofenac (5-10/month)
3	49	120	1.0	2	2	2	2	Ibuprofen (2-3/month)
4	52	88	3.0	3	3	9	5	Diclofenac (0-8/month)
5	49	240	2.2	5	8	4	5	Ibuprofen (0-1/month)
6	54	108	4.6	3	6	6	3	Diclofenac (2-3/month)
7	50	> 60	3.5	5	7	11	5	Flupirtin (1-10/month)
8	56	> 60	3.5	4	7	9	6	No medication at all
9	56	> 60	1.1	4	7	11	2	Diclofenac (0-10/month)
10	44	> 60	1.0	1	1	7	2	Ibuprofen (0-3/month)
11	55	147	3.6	3	5	4	5	No medication at all
12	54	> 60	3.1	5	6	7	3	Ibuprofen (0-10/month)
13	48	> 60	3.8	2	4	2	6	No medication at all
14	56	> 60	1.0	2	3	11	1	No medication at all
CLBP (mean ± SD)	52.1 ± 3.7	> 60**	3.0 ± 1.7**	3.4 ± 1.4**	5.3 ± 2.3**	7.6 ± 3.5**	4.7 ± 3.4**	
HC (mean ± SD)	51.9 ± 4.9	0.0 ± 0.0	0.0 ± 0.0	0.0 ± 0.0	0.0 ± 0.0	1.8 ± 1.5	0.1 ± 0.5	

VAS^QST^, 100 mm visual analog scale (VAS): current pain intensity before Quantitative sensory testing (QST); NRS ^4average^, numerical rating scale (NRS) pain intensity rating in response: “How would you rate your *average* pain over the last four weeks?”; NRS ^4max^, numerical rating scale (NRS) pain intensity rating in response: “How would you rate your *highest* pain intensity experienced within the last four weeks?” VAS/NRS left anchor with 0 = “no pain” and right anchor with 100/10 = “pain as bad as you can imagine”; BDI – Beck Depression Inventory [28]; RDQ – Roland and Morris Disability Questionnaire [63–65]. Reported medications: Mean use of medication; however, all CLBP patients were without any analgesic medication for at least 48 hours before the examination. All participants were employed. Significant difference between CLBP and HC: ** - P < 0.001.

To evaluate our hypothesis, pain sensitivity to punctate stimuli was tested unilaterally on the most painful body site (paraspinal lumbar; location of measurement: vertebra Th12 to L5) and on two non-painful body sites (hand palmar, hand dorsum; unilaterally on the dominant right hand) in CLBP patients. Healthy control subjects were matched on age and gender to the patients (see Table 1). Testing in healthy control subjects was performed at the same regions on the lower back and the dominant hand.

### Stimulus response function (SRF) to punctate mechanical stimuli

In accordance with our hypothesis, we used a test for mechanical pain thresholds from the Quantitative sensory testing (QST) according to the standardized protocol of the German Research Network on Neuropathic Pain (DFNS) [26,27], i.e., the stimulus response function for pinprick stimuli that also allows mechanical pain sensitivity (MPS), another parameter of QST, to be determined. Testing was performed by a DFNS-trained investigator.

*Stimulus response functions (SRF) to punctate mechanical stimuli* were analysed using standard pinprick stimulators (cylindrical tip, 250 μm tip diameter) with fixed stimulus intensities that exerted forces of 8, 16, 32, 64, 128, 256, and 512 mN (MRC Systems GmbH, Heidelberg, Germany). All seven pinprick stimuli were applied in a balanced order, five times each at every test site (back paraspinal lumbar, hand palmar, hand dorsum). SRF was assessed as the relationship between the applied forces of the standard pinprick stimulators and the pain rating evoked by each of the pinprick stimulators [28,29], i.e., participants were asked to rate the experienced pain intensity after each stimulation on a verbal rating scale (with 0 indicating “no pain”, and 100 indicating “maximal imaginable pain”). To avoid effects of sensitization or fatigue, successive stimuli were not applied at the same spot of skin, but some millimetres away from the preceding stimulation site.

*Mechanical pain sensitivity (MPS)* was also determined. MPS was assessed as the geometric mean of the pain ratings evoked by each of the seven pinprick stimuli (similar to the standard protocol of DFNS).

In order to exclude a potential influence of skin temperature on the results of testing, skin temperature was assessed for all body sites (back paraspinal lumbar, hand palmar, hand dorsum) before and after the testing. There were no significant skin temperature changes for any of the body sites before vs. after the testing or between patients and controls.

### Questionnaires

Depression was assessed using a German version [30] of the Beck Depression inventory (BDI) [31]. None of the participants suffered from clinically manifest depression (cut-off BDI > 17; Table 1).

Disability was measured using the German version of the 24-item Roland Morris disability questionnaire (RDQ) [32-34]. RDQ scores range from 0 (no disability) to 24 (maximum disability). Five of the CLBP patients showed clinically-relevant disability (3 < RDQ score < 8); 1 patient reported a high level of disability (RDQ > 7); none of the control subjects reported clinically-relevant disability (Table 1).

### Data evaluation

The experienced pain intensities of each of the seven pinpricks (SRF) as well as MPS were normally distributed in log space, and thus were log_10_-transformed before statistical analysis in accordance with the recommendations of DFNS [27]. For SRF and MPS, a small constant (0.1) was added prior to log-transformation to avoid a loss of values due to zero rating [35]. Data transformed to secondary normal distribution were analysed with analysis of variance (ANOVA) for repeated measurements using the within-subject factors Pinprick (7 intensities from 8 to 512 mN) and Region (hand dorsum vs. hand palmar vs. lower back) as well as the between-subject factor Group (CLBP vs. HC). Post-hoc tests were performed using separate ANOVAs. Results were corrected for violations of sphericity using the Greenhouse-Geisser approach for epsilon correction of degrees of freedom (when appropriate). All statistical calculations were performed using SPSS 19 Software.

Data were z-transformed to compare the MPS with healthy control data and the data of the DFNS for the hand dorsum using the following expression:

(1) $$Z-score=\frac{\left(value_{individual\;CLBP\;patient}-mean_{\mathrm{controls}}\right)}{SD_{\mathrm{controls}}}$$

MPS for each CLBP patient was compared with the group means of healthy controls using z-scores. Z-scores above ‘0’ indicate a gain of function referring to the higher sensitivity of the CLBP patient to the tested stimuli compared to the healthy controls. Z-scores below ‘0’ indicate a loss of function when the CLBP patient is less sensitive to the tested stimuli compared with the healthy controls. Z-values below −1.96 or above +1.96 were considered as abnormal for diagnostic purposes (95% confidence interval [33,34]). A *t*-test (two-sided for independent samples) was performed for MPS using the internet-based statistical freeware Simple Interactive Statistical Analysis (SISA; URL: http://www.quantitativeskills.com/sisa/) separately for healthy controls and CLBP patients to compare data with the DFNS reference data as proposed recently [33].

## Results

### Detailed analysis of SRF to pinprick stimuli

**Figure 1:**
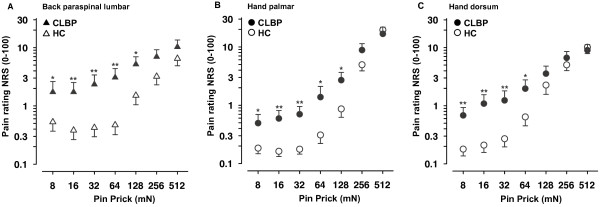
**Stimulus response functions to pinprick stimuli on the back and dorsal and palmar aspects of the hand.** Psychometric stimulus response functions (SRF) for pain to punctate probes of different forces are presented for **A**. the paraspinal lumbar test site (back, triangles) and the remote control sites, **B**. hand palmar (circles), and **C**. dorsum of the hand (circles). Mean ± SEM of the log-transformed values are shown. Filled symbols: patients with chronic low back pain (CLBP), open symbols: healthy controls (HC). Significant difference between CLBP and HC: *- P < 0.05; ** - P < 0.01.

ANOVA on pain ratings to pinprick stimulation with factors Pinprick, Region, and Group showed significant main effects of factors Pinprick (F(6, 156) = 187.02; P < 0.001), Region (F(2, 52) = 11.57; P < 0.001), and Group (F(1, 26) = 7.912; P < 0.01). As expected, the main effect of factor Pinprick resulted from higher ratings to pinpricks with higher force (see Figure 1A-C). The main effect of factor Region resulted from overall higher pain ratings at the dorsum of the back compared with the hand (hand palmar vs. back: F(1, 26) = 16.62, P < 0.001; hand dorsum vs. back: F(1, 26) = 8.38, P < 0.01; hand palmar vs. hand dorsum: F(1, 26) = 5.99, P < 0.05). The main effect of Group is explained by overall higher pain ratings in the CLBP group as compared to HC (F(1, 26) = 7.91, P < 0.01, Cohen’s d = 1.44; see Figure 1A-C). Additionally, there were significant interactions between the factors Pinprick x Region (F(12, 312) = 16.53, ε = 1.63; P < 0.001) and Pinprick x Group (F(6, 156) = 9.07, ε = 3.28; P < 0.001). One-way ANOVAs for the interaction Pinprick x Group revealed significant differences in pain ratings between groups for the pinpricks 8 mN, 16 mN, 32 mN and 64 mN (all P < 0.05, corrected for multiple testing; all d > 1; range: 1.06 – 1.40). The perceived pain in CLBP patients was 3–5 times higher for low to moderate pinprick forces compared with healthy controls. The difference in pain rating between groups for the 128 mN pinprick was significant at an uncorrected level (P < 0.037, d = 0.83), whereas no difference in pain ratings between groups was observed for the 256 mN (P = 0.117, d = 0.61) and 512 mN pinpricks (P = 0.849; d = 0.08) (see Figure 1A-C). No significant interactions other than Pinprick x Group and Pinprick x Region were observed. In particular, there was no 3-way Pinprick x Region x Group interaction (F(12, 312) = 1.46, ε = 6.28; P = 0.19) indicating that the observed differences between groups and the interaction between Pinprick and Group were not different for hand and back.

### Detailed analysis of MPS

**Figure 2:**
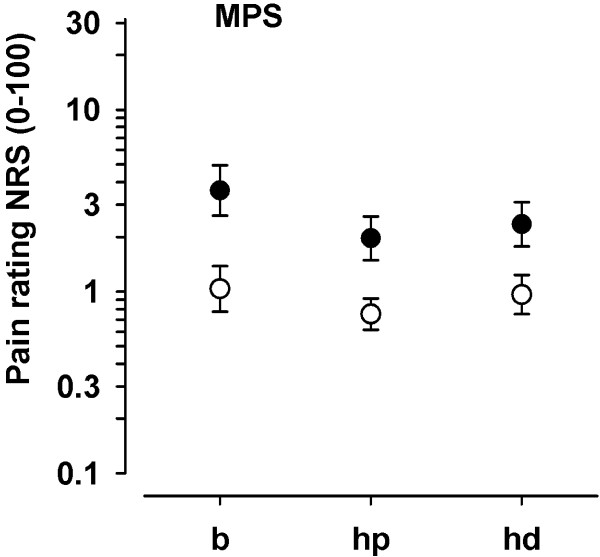
**Mechanical pain sensitivity (MPS) of the back and dorsal and palmar aspects of the hand.** MPS (mean ± SEM) of patients with chronic low back pain (filled circles) and healthy controls (open circles) is depicted for the paraspinal lumbar back region (b), hand palmar (hp), and dorsum of the hand (hd). Note that MPS differed between groups for all regions under investigation.

ANOVA of MPS revealed main effects of factors Region (F(2, 52) = 11.63, P < 0.001) and Group (F(1, 26) = 7.90, P < 0.01), but no interaction between Region and Group (F(2, 52) = 1.93, P = 1.55. As Figure 2 shows, the main effect of factor Region resulted from significantly higher MPS for the back compared with the hand (back vs. hand palmar: F(1, 26) = 16.78, P < 0.001; back vs. hand dorsum: F(1, 26) = 8.41, P < 0.01; hand dorsum vs. hand palmar: F(1, 26) = 6.01, P < 0.05). The main effect of factor Group is explained by overall higher MPS in the CLBP group as compared to HC (F(1, 26) = 7.90, P < 0.01, d = 1.06; see Figure 2). MPS of the palmar site of the hand as well as the dorsum of the hand were highly correlated with the MPS at the back (z-score based correlations, hand palmar: R^2^ = 0.78, P < 0.001; hand dorsum: R^2^ = 0.83, P < 0.001). Furthermore, MPS of the palmar site and of the dorsum of the hand were also highly correlated (R^2^ = 0.81, P < 0.001).

### Incidence of reported pain to punctate stimuli

**Figure 3:**
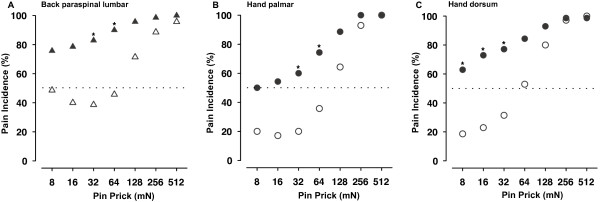
**Incidence of the reported pain to pinprick stimuli on the back and palmar and dorsal aspects of the hand.** Incidence of reported pain is shown as a function of the pinprick stimulus force **A**. at the back (triangles), **B**. hand palmar (circles), and **C**. the dorsum of the hand (circles). Filled symbols: patients with chronic low back pain (CLBP), open symbols: healthy controls (HC). Population threshold (force at which 50% of pinprick stimuli were reported to be painful): dotted line in A, B and **D**. Asterisks in A, B, C depict significant differences (P < 0.05) between groups in the incidence of reported pain.

Figure 3 shows that the incidence of reported pain increased as a function of stimulus force on the back, hand palmar, and hand dorsum (Figure 3A-C, respectively). The population threshold, i.e., force at which 50% of pinprick stimuli were reported to be painful, was interpolated as 8 mN for the CLBP patients and 96 mN in healthy controls at the back. Additionally, there were significant differences between groups in the incidence of reported pain on the back for 32 mN (*χ*^2^ = 12.07; p < 0.05) and 64 mN (*χ*^2^ = 10.91; p < 0.05) (see Figure 3A). On the hand palmar, the population threshold was also interpolated as 8 mN for the CLBP patients and 96 mN in healthy controls. There were significant differences between groups in the incidence of reported pain at 32 mN (*χ*^2^ = 11.03; p < 0.05) and 64 mN (*χ*^2^ = 11.4; p < 0.05) on the hand palmar (see Figure 3B). For the dorsum of the hand the population threshold was interpolated as 8 mN for the CLBP patients and 48 mN in healthy controls. There were significant differences between groups in the incidence of reported pain at 8 mN (*χ*^2^ = 12.92; p < 0.05), 16 mN (*χ*^2^ = 12.48; p < 0.05) and 32 mN (*χ*^2^ = 10.60; p < 0.05) on the hand dorsum (see Figure 3C).

### Comparison to reference data

We also compared our MPS data (detailed data on SRF are not publicly available in the DFNS reference data) with respect to the reference data from the German Research Network on Neuropathic Pain (DFNS). First, we compared the results of our HC group with the normative data. Results obtained at the hand of our HC group were in the range of the DFNS reference data as judged by the recommended quality self-control procedure (average z-score of 0.09 for HC subjects was < 95% confidence interval of DFNS reference data, recommended to be < 0.25; [36]) indicating a good agreement with the DFNS standard. The data of our HC group was almost perfectly matched to the reference data (t = 0.05, P = 0.62). Second, *T-tests* confirmed a significant group difference for MPS on the hand dorsum for the CLBP patients with respect to our HC group on the basis of z-transformed values (t = 2,96 P < 0.05) as well as with respect to the reference data (t = −2.65, P < 0.05). Furthermore, the MPS z-scores for the hand dorsum of CLBP patients normalized to our designated matched control group, and the z-scores normalized to the DFNS reference data [36], were significantly correlated (R^2^ = 0.979, P < 0.001) underlining the robustness of results with respect to reference data.

## Discussion

The aim of the present study was to investigate the stimulus response function to punctate mechanical pinprick stimuli in female CLBP patients on the affected painful region (lumbar) and at an extraterritorial region (dominant hand). CLBP patients exhibited an enhanced sensitivity and higher pain ratings to punctate mechanical pinprick stimuli, especially pronounced at low to moderate stimulus intensities. Importantly, significantly enhanced pain sensitivity to pinpricks, a hallmark of central sensitization in experimental models and in neuropathic pain, was identified in both the paraspinal lumbar area and the dorsal and palmar surfaces of the hand in CLBP patients compared with matched healthy controls.

### Stimulus response function to punctate pinprick stimuli at the back

Our data revealed significant changes on the back for punctuate mechanical pinprick stimuli, especially pronounced at low to moderate stimulus intensities. SRFs (Figure 1A) showed manifold increases of pain ratings for low to moderate intensities compared to healthy controls. SRFs converged for higher intensities. Compared to healthy controls pain ratings to pinpricks were increased by a factor of 2 to 6. Beyond the frequent finding of muscle pain thresholds, this is the first report demonstrating hyperalgesia to pinprick stimuli, specifically the pronounced allodynia to low-intensity pinprick stimuli in CLPB patients. These changes are a hallmark of centrally mediated hyperalgesia, and are found in experimental models of central sensitization (neurogenic hyperalgesia [29,36-40] reviewed in [41,42]) as well as in neuropathic pain patients [43-46]. Specificity for facilitation of this stimulus modality has also been shown in animal models of central sensitization at the level of the spinal dorsal horn, thalamus, and amygdala [47-50].

### Stimulus response function to punctate pinprick stimuli on the hand

Interestingly, we also found significant changes to punctate pinprick stimuli on the hand (MPS, stimulus response function), especially pronounced at low to moderate stimulus intensities (see SRF and pain incidence in Figures 1B-C, 3B-C) in CLBP patients. This finding may be of principal importance because it demonstrates changes in pain sensitivity extending far beyond the painful lumbar back. More specifically, there was no significant effect of factor Region suggesting that the increase in sensitivity in CLBP patients to mechanical punctate stimuli is similar for the back and the hand. The mechanisms for this widespread pain sensitivity are largely unknown. Potential explanations include e.g. altered attentional processes, loss of inhibitory pain control, or higher vulnerability in CLBP patients.

A further potential explanation may be related to plasticity at supraspinal levels, for which the thalamus is a prime candidate, since it forms the next relay of the ascending pathway. Receptive field sizes in the thalamus are much larger than in the spinal cord, both in the specific pathways of lateral thalamus projecting to the somatosensory cortices, and in the non-specific pathways of medial thalamus projecting to the anterior cingulate cortex and amygdalae, where receptive field sizes can encompass whole quadrants, or body sides [51]. Several findings in animals support the involvement of the thalamus. In one such study, facial mechanical allodynia spreading from the affected to the contralateral side was accompanied by enhanced thalamic transmission [52]. Contralateral spread of mechanical hyperalgesia and allodynia also appears in more severe cases of migraine in human patients [47,52]. Similar mechanisms have been found in nociceptive plasticity of the spinal or medullary dorsal horn and thalamic plasticity [53,54]. The modality specificity of experimentally-induced mechanical hyperalgesia was also reported as preserved in the amygdala receiving ascending projections from the medial thalamus, as well as descending input from the anterior cingulate cortex and insula [48]. Data on extrasegmental spread however are not yet published. Nevertheless, reverse modulation has been demonstrated in patients with fibromyalgia after two terms of successful local treatment of tender points with widespread relief of signs of hyperalgesia [55].

Alternatively, a relative insufficiency of descending control may passively, or descending facilitation may actively, produce an expansion of receptive field sizes both segmentally and extrasegmentally. Human and animal data have demonstrated that experimentally-induced secondary hyperalgesia involves descending facilitation and spreads rapidly into neighbouring segments [49,56]. However, there is no animal data so far demonstrating that sustained spinal nociceptive input may spread sensitization for more than a few segments, e.g. from lumbar to cervical level. Another alternative explanation for the hypersensitivity might be an a priori increased sensitivity to mechanical stimuli in CLBP patients compared to HC. Longitudinal approaches might test this assumption.

### Limitations and further directions

Our sample size of 14 subjects in each group is relatively small, so the study should be extended to larger sample sizes and different centres. Furthermore, we only tested female subjects, whereas previous studies have demonstrated gender differences in pain thresholds (Magerl et al. 2010). Our study should therefore be extended to the investigation of SR functions in male subjects. Additionally, we did not follow the menstrual cycle of our participants although there are known variations in pain perception during the cycle. Nevertheless, in our small sample, the sensory changes we report are robust and the effect sizes large (CLBP vs. HC, MPS back: d = 1.08; MPS hand dorsum: d = 0.90; MPS hand palmar: d = 1.07).

The data do not allow us to distinguish between the two major hypotheses for this higher-order effect, i.e., sensitization after an aversive event vs. susceptibility. Longitudinal studies might help to solve this question. Alternatively, identifying subjects with a specific pinprick hyperalgesia without CLBP (or other chronic pain symptoms) might be informative about the independence of the observed phenomena.

## Conclusions

In summary, we have found a widespread hypersensitivity to punctate mechanical pinprick stimuli in CLBP patients compared to HC. To our knowledge, this is the first study reporting such a widespread hypersensitivity with a 2 to 6-fold increase in pain ratings for low to moderate pinprick stimuli in CLBP patients. This kind of specific pinprick hyperalgesia was found not only in the affected painful site (back), but also in the remote reference site on the hand. This result points to higher-order plasticity in CLBP or higher vulnerability rather than to restricted spinal cord mechanisms.

## Abbreviations

CLBP: Chronic Low Back Pain; HC: Healthy Control Subjects; DFNS: German Research Network on Neuropathic Pain; QST: Quantitative Sensory Testing; MPS: Mechanical Pain Sensitivity; SRF: Stimulus Response Function.

## Competing interest

All authors declare no competing of interests.

## Authors’ contributions

CP conceived of the study and developed the design, supervised and participated in data acquisition and carried out data analysis, interpretation and drafted the manuscript. BS carried out the acquisition of the subjects, data acquisition, and participated in data analysis. SS carried out data acquisition and participated in data analysis. WM has been involved in interpretation and discussion of data and drafting the manuscript. BG participated in acquisition of the patients and data analysis. HHWG participated in the conception of the study and in drafting the manuscript. WHRM participated in the conception of the study and in drafting the manuscript. TW conceived of the study, participated in the development of its design, coordinated part of the examination, and participated in drafting the manuscript. All authors read and approved the final manuscript.

## Funding

This work was supported by the Federal Ministry of Education and Research BMBF [01EC1003, 01EC1010].

## Pre-publication history

The pre-publication history for this paper can be accessed here:

http://www.biomedcentral.com/1471-2377/12/98/prepub
